# Crosstalk Correction for Color Filter Array Image Sensors Based on *L*_p_-Regularized Multi-Channel Deconvolution

**DOI:** 10.3390/s22114285

**Published:** 2022-06-04

**Authors:** Jonghyun Kim, Kyeonghoon Jeong, Moon Gi Kang

**Affiliations:** School of Electrical and Electronic Engineering, Yonsei University, Seoul 03722, Korea; jhyun15@yonsei.ac.kr (J.K.); qazwxd91@yonsei.ac.kr (K.J.)

**Keywords:** crosstalk phenomenon, crosstalk correction, color filter array, multi-channel deconvolution, *L_p_* regularization

## Abstract

In this paper, we propose a crosstalk correction method for color filter array (CFA) image sensors based on $L_{p}$-regularized multi-channel deconvolution. Most imaging systems with CFA exhibit a crosstalk phenomenon caused by the physical limitations of the image sensor. In general, this phenomenon produces both color degradation and spatial degradation, which are respectively called desaturation and blurring. To improve the color fidelity and the spatial resolution in crosstalk correction, the feasible solution of the ill-posed problem is regularized by image priors. First, the crosstalk problem with complex spatial and spectral degradation is formulated as a multi-channel degradation model. An objective function with a hyper-Laplacian prior is then designed for crosstalk correction. This approach enables the simultaneous improvement of the color fidelity and the sharpness restoration of the details without noise amplification. Furthermore, an efficient solver minimizes the objective function for crosstalk correction consisting of $L_{p}$ regularization terms. The proposed method was verified on synthetic datasets according to various crosstalk and noise levels. Experimental results demonstrated that the proposed method outperforms the conventional methods in terms of the color peak signal-to-noise ratio and structural similarity index measure.

## 1. Introduction

Advancements in image acquisition devices, including camera imaging systems, have continually opened new application areas. In recent years, the rapid development of imaging systems fostered by mobile-camera technology has promoted the application of imaging systems in many fields, such as security, military, aerial imaging, satellite imaging, and autonomous vehicles. Accordingly, the demand for cost-effective high-performance imaging using low computational power has increased. To fulfill this need for efficiency in camera imaging systems, a single sensor based on a color filter array (CFA) is essential, instead of using three sensors or optical beam splitters.

In response to consumer perceptions and expectations of digital color image contents, attempts have been made to increase the spatial resolution engendered by the number of pixels in a limited-sized imaging system. Many signal-processing-based methods for single sensor architectures have been required to achieve high-resolution, high-sensitivity imaging. Studies on CFAs [1,2,3] and demosaicing methods [4,5,6,7,8] have been conducted to maximize CFA performance. To increase the spatial resolution in a limited-size image sensor, the pixel size should be reduced. However, this approach can compromise the sensitivity [9]. As a result, smaller pixels tend to generate more noise. Thus, denoising algorithms [10,11] are needed to solve such problems, which often occur in low-light situations. Although the effect on noise is minimized through signal processing, the issue caused by the pixel size reduction remains.

**Figure 1:**
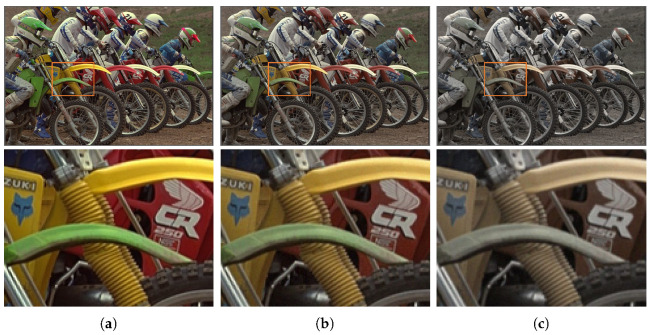
Problems of imaging under the crosstalk condition in CFA: (**a**) Original image; (**b**) degraded image by Gaussian kernel with $\sigma_{g}=0.45$ and noise with $\sigma_{n}=0.01$; (**c**) degraded image by Gaussian kernel with $\sigma_{g}=0.6$ and noise with $\sigma_{n}=0.01$. As the standard deviation $\sigma_{g}$ of the Gaussian kernel increases, desaturation and blurring intensify owing to interference with neighboring channels.

Another problem caused by the pixel size reduction is the crosstalk phenomenon. Specifically, it is caused by the physical limitations of the image sensor, that is, the leakage of photons and electrons between adjacent pixels [12,13]. As the pixel size continues to shrink, crosstalk between adjacent pixels emerges. In CFA image sensors, photon and electron leakage due to optical refraction and minority carriers inevitably occurs as the geometric distance between adjacent pixels decreases. In the obtained image, crosstalk typically generates two artifacts: desaturation and blurring. As shown in Figure 1, color constancy and preservation of the spatial resolution of the image thus cannot be achieved.

Several attempts have been made to overcome this crosstalk phenomenon. The first approach is based on the hardware design of the sensor or CFA. To achieve better color reproduction, the CFA is designed to optimize the spectral responses of the entire imaging system by compensating for the crosstalk effects [14]. Use of backside illumination technology with a parabolic color filter decreases the crosstalk while increasing the efficiency [15]. New CFA patterns have been proposed to minimize crosstalk in a sensor with a small pixel size using a combination of primary and complementary color filters, such as yellow, cyan, and magenta, as in [12]. Although the crosstalk effect has been reduced through physical improvements, the image sensors experience difficulties as the pixel size decreases.

The second approach is based on signal processing. For example, the crosstalk phenomenon was analyzed by Hirakawa et al. using a spatio-spectral sampling theory, which was formulated by using a convolution operation [13]. This method assumes that the most significant artifact is desaturation; thus, it focuses on color correction. However, the color correction method that employs matrix inversion may have limitations owing to certain issues, such as blurry artifacts and noise amplification under low-light conditions.

To solve the desaturation and blurring problem, joint decrosstalk and demosaicing method was proposed [16]. This method is based on a piecewise autoregressive image model. It performs demosaicing iteratively under crosstalk constraints. However, this method is not practicable because it is difficult to utilize recently studied demosaicing algorithms, such as ARI [8], and it requires considerable computation. In addition, because this method focuses more on estimating the edge directional weight that is required for demosaicing, it does not sufficiently preserve the edges in constraint. Therefore, we herein propose a crosstalk correction method that is based on multi-channel deconvolution with a hyper-Laplacian prior. It restores the image spatial resolution and color fidelity without producing obvious artifacts.

The main contributions of this study are the following:

- The crosstalk problem is formulated as a multi-channel degradation model;
- A multi-channel deconvolution method based on the objective function with a hyper-Laplacian prior is designed. The proposed method utilizes $L_{p}$ regularization to achieve the estimated image with sharp edges and details, and it efficiently suppresses noise amplification for each color component. Concurrently, intercolor regularization is employed to smooth the color difference components and to encourage the homogeneity of the edges;
- An efficient algorithm based on alternating minimization is described. Experimental results validate that the proposed method is more robust than conventional methods.

The remainder of this paper is organized as follows. Section 2 presents the crosstalk analysis and problem formulation on the multi-channel degradation model. In Section 3, the proposed method with a hyper-Laplacian prior is detailed. Section 4 presents the experimental results. Finally, Section 6 concludes this paper.

## 2. Problem Formulation

An image sensor is based on either a charge-coupled device (CCD) or a complementary metal-oxide-semiconductor (CMOS). By using CFA and a microlens array, the image sensor converts the light received from the main lens into digital signals. As each color filter has its own spectrum, only one piece of color information is obtained from one pixel.

To estimate an original color image from a subsampled image according to the CFA pattern, demosaicing is used. It is intended to overcome the physical limitations of a single-sensor imaging system. Consider an original discrete color image, $x\left(n\right)=[x_{R}\left(n\right),x_{G}\left(n\right),x_{B}\left(n\right)]$, where $n\in Z^{2}$, and $x_{R}\left(n\right)$, $x_{G}\left(n\right)$, and $x_{B}\left(n\right)$ are the three color components for respective red (R), green (G), and blue (B) channels. The subsampled image $y_{s}\left(n\right)$ according to the CFA pattern can be expressed as

(1) $$y_{s}\left(n\right)=\sum_{k\in \{R,G,B\}}d_{k}\left(n\right)x_{k}\left(n\right)+e\left(n\right),$$

where $d_{k}\left(n\right)$ is the subsampling function of CFA and k∈{R,G,B}. This function is periodic, and $d_{R}\left(n\right)+d_{G}\left(n\right)+d_{B}\left(n\right)=1$ for each pixel. The term e(n) is the corresponding signal-independent additive noise.

**Figure 2:**
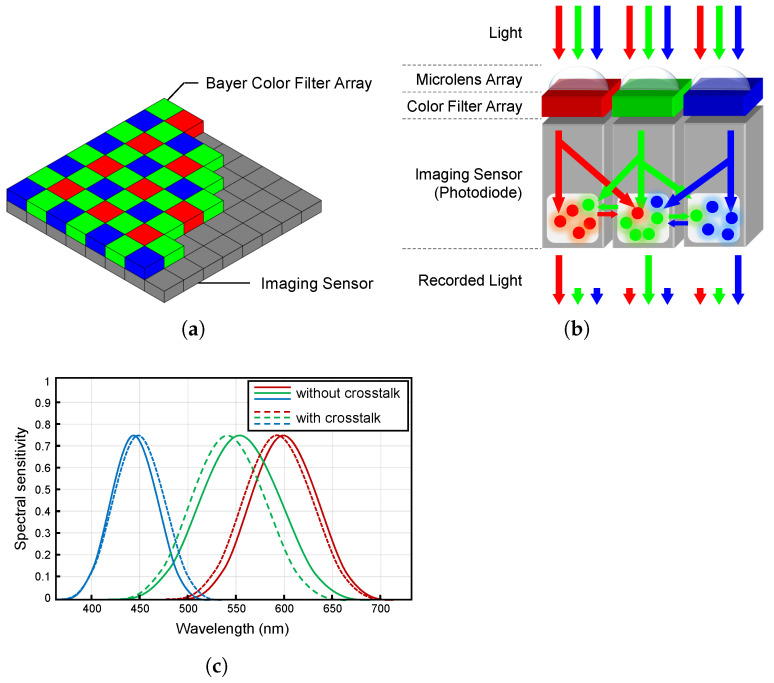
Bayer CFA and characteristics of crosstalk phenomenon: (**a**) Bayer CFA structure. The Bayer CFA consists of three filters for respective R, G, and B channels. (**b**) Schematic image of crosstalk inside the imaging sensor. (**c**) Example of the spectral sensitivity of the camera imaging system in a crosstalk-free condition (solid lines) and in a crosstalk condition (dashed lines). The spectral sensitivity shift is caused by crosstalk inside the imaging sensor.

During the process of obtaining a subsampled image, desaturation and blurring occur as a result of the crosstalk phenomenon. Figure 2 illustrates the structure of the imaging system. The light entering through the camera’s main lens sequentially passes through the microlens and color filter and reaches the imaging sensor. In this process, interference occurs on account of optical diffraction and minority carriers caused by the surrounding pixels. Therefore, the image acquired by the imaging sensor suffers simultaneously from spatial and spectral degradation. As shown in Figure 2c, the spectral sensitivity shifts owing to crosstalk inside the imaging sensor. The observation model with the crosstalk phenomenon can be represented as follows:

(2) $$\begin{array}{cc} y_{ct}\left(n\right) & =\sum_{m}b\left(n,m\right)y_{s}\left(n\right)+e\left(n\right)\\ \; & =\sum_{m}\sum_{k\in \{R,G,B\}}b\left(n,m\right)d_{k}\left(n\right)x_{k}\left(n\right)+e\left(n\right), \end{array}$$

where $y_{ct}\left(n\right)$ is the subsampled image under the crosstalk phenomenon and b(n,m) is the crosstalk function. This function represents a combination of optical diffraction and minority carriers. We assume that the crosstalk function is space-invariant. Using matrix notation, the relation between the subsampled image and the original image is rewritten as follows:

(3) $$y_{ct}=BDx+e,$$

where x denotes the three color component vectors of the original color image with *M* rows and *N* columns; that is, $x^{T}=\left[x_{R}^{T},x_{G}^{T},x_{B}^{T}\right]$. Moreover, y denotes the observed image acquired by the sensor according to the CFA, and e is corresponding additive noise. Vector x has dimensions 3MN, and each vector y and e is a vector of dimensions MN. Matrix B has dimensions MN×MN and denotes a crosstalk function, while D is a subsampling operator with dimensions MN×3MN.

In general, it is important to solve the inverse problem according to the degradation model. If the observation model of Equation (3) is a linear shift-invariant (LSI) system, BD can be replaced with DB since the commutative property is established. The special type of matrix associated with LSI systems is known as the Toeplitz matrix. In the above system, B satisfies this matrix; however D is not a Toeplitz matrix. In other words, subsampling operator D violates the commutative property of convolution. Thus, to solve the Equation (3) problem, the inverse problem with respect to matrix D should be solved sequentially after solving the inverse problem in terms of matrix B and considering noise e.

On the other hand, the crosstalk degradation is solved by focusing on color fidelity in [13] after solving the D matrix, which relates to subsampling through demosaicing. As this method performs demosaicing first, it is not suitable for handling the modeling presented in Equation (3). Alternatively, a method of performing deconvolution ($B^{-1}$) in the subsampled domain should be considered prior to demosaicing ($D^{-1}$). It is important to solve the problems sequentially according to modeling. However, performing deconvolution in the subsampled domain causes another problem. Although the method can solve the blur phenomenon in Equation (3), it does not sufficiently separate mixing between color channels. Furthermore, an iterative approach is used that repeatedly performs demosaicing and deconvolution in the subsampled domain to overcome blur artifacts and maintain color fidelity in [16].

**Figure 3:**
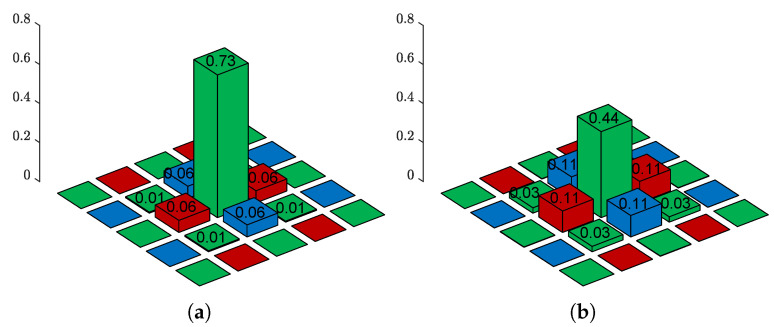
Crosstalk kernels at G location: (**a**) Gaussian kernel of $\sigma_{g}=0.45$ and (**b**) Gaussian kernel of $\sigma_{g}=0.60$. Owing to the crosstalk kernel, spectral and spatial degradation simultaneously occur.

The crosstalk function is not a simple low-pass filter. Rather, it is a complex process in which the spectral information of three color components is mixed and the spatial information of the surrounding pixels are simultaneously mixed in the subsampled domain, as shown in Figure 3. In particular, as the standard deviation of the Gaussian kernel increases, the image degradation due to the crosstalk phenomenon increases. As the noise increases, it becomes difficult to improve the color fidelity. Therefore, a new formulation is needed to solve the problem based on a multi-channel degradation model. The following multi-channel degradation model is considered:

(4) y=DHx+e,

where the condition BD=DH should be satisfied, and multi-channel degradation matrix H is defined by

(5) $$H=\left[\begin{array}{ccc} H_{RR} & H_{RG} & H_{RB}\\ H_{GR} & H_{GG} & H_{GB}\\ H_{BR} & H_{BG} & H_{BB} \end{array}\right].$$

Here, $H_{kl}$ is the dimension MN×MN for (k,l)∈{R,G,B}. Diagonal matrices $H_{kk}$ represent the within-channel kernel, and off-diagonal matrices $H_{kl}$ represent the cross-channel kernel when k≠l.

**Figure 4:**
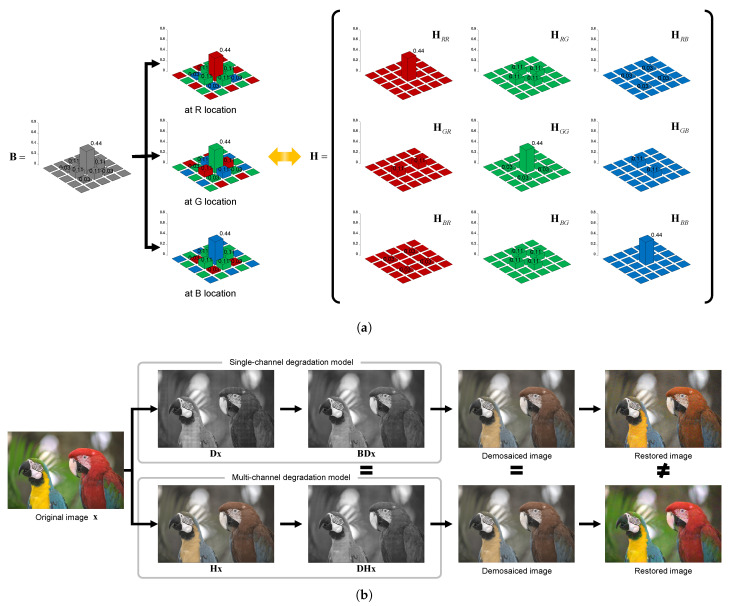
Example illustrations of single-channel and multi-channel degradation models: (**a**) Relationship of B in the single-channel degradation model (left) and H in the multi-channel degradation model (right). (**b**) Comparison of the single-channel degradation model (top) and the multi-channel degradation model (bottom). ARI demosaicing is applied equally to degraded images. The same degraded results are produced by different formulations of the crosstalk phenomenon, but different results are restored.

Matrix H can be composed by utilizing the characteristic that each location and channel has a different kernel according to the CFA periodic structure. Figure 4a shows an example of configuring matrix H using matrix B and the CFA structure. As each color filter has its own spectrum, the crosstalk kernel is separated into three different kernels depending on the location of each color filter even though they have the same shape as the low-pass filter. Thereafter, a total of nine kernels can be configured by separating each color channel. Note that multi-channel degradation matrix H changes according to the type of CFA pattern. In the case of a RGBW [3] or multispectral filter array [2], the number of spectral bands and spatial periodicity of CFA sampling patterns are different and the formulation thus must be slightly changed.

Figure 4b shows that different formulations—single-channel and multi-channel degradation models—can yield the same degraded results. By modeling the same phenomenon differently, we propose a method that can simultaneously solve complex spatial and spectral degradation through a multi-channel degradation model.

## 3. Proposed Method

In this section, we propose an efficient algorithm for multi-channel deconvolution based on the multi-channel degradation model, as mentioned above. To overcome the ill-posed problem, effective regularization terms are employed to restrict the solution space. The proposed method utilizes $L_{p}$ regularization to achieve the estimated image with sharp edges and details. It efficiently suppresses noise amplification for each color component. Concurrently, intercolor regularization is employed to smooth the color difference components and to encourage the homogeneity of the edges. Thereafter, we describe an efficient solver to minimize the cost function regularized by specific prior information.

### 3.1. Multi-Channel Deconvolution

The multi-channel deconvolution problem is used to obtain an estimate of x given y, D, H, and the prior knowledge of e. Equation (4) can be rewritten as

(6) $$\begin{array}{c} y_{k}=D_{k}H_{k}x+e_{k},\;\;\mathrm{for}\;k\in \left\{R,G,B\right\}, \end{array}$$

where matrix $H_{k}$ is given by

(7) $$H_{k}=\left[H_{kR},H_{kG},H_{kB}\right],$$

with the *k*th block row matrix of dimension MN×3MN. The regularized solution of the multi-channel deconvolution problem in (6) is defined as

(8) $$\hat{x}=\arg\min_{x}\left\{\sum_{k\in \{R,G,B\}}\frac{1}{\lambda_{k}}\left|\right|y_{k}-D_{k}H_{k}x{\left|\right|}_{2}^{2}+R\left(x\right)\right\}.$$

Here, the first term, called the data fidelity term, denotes a measure of the Euclidean distance from the original image to the observed image. The second term R(x) is the regularization term based on the prior model. Regularization parameters $\lambda_{k}$ manage the trade-off between the two terms. As each color channel suffers from degradation under the same conditions, it is assumed that $\lambda_{k}$ for each channel has the same value.

The data fidelity term can generally be defined as the $L_{2}$-norm, which is known as the least-squares approach. Although the $L_{1}$-norm increases the robustness to outliers such as noise or errors, it is employed in this study because the error term is generally modeled as Gaussian noise and the crosstalk kernel is an already known prior. As the crosstalk kernel is not signal-dependent, it can be parameterized through experiments. Moreover, it is generally assumed to be a Gaussian kernel [14]. In addition, $L_{2}$-norm data fidelity enables the finding of solutions with low computational costs [17].

The regularization term is herein adopted to decrease the uncertainty in the inverse problem by constraining the solution space. The regularization term can be expressed according to the combination of several constraints:

(9) $$R\left(x\right)=\sum_{i}\lambda_{i}R_{i}\left(x\right),$$

where $\lambda_{i}$ is the regularization parameter for each constraint function $R_{i}\left(x\right)$. Tikhonov regularization is a representative smoothing constraint that assumes that the distribution of gradient magnitudes for images is smooth [18]. This strategy decreases the uncertainty using the $L_{2}$-norm and is defined as follows:

(10) $$R\left(x\right)={\left||Cx|\right|}_{2}^{2},$$

where C denotes the second derivative linear operator, which is known as the Tikhonov matrix. Tikhonov regularization smooths the estimated image with a limited high-frequency component. However, this regularization strategy inevitably oversmooth some important information, such as edge sharpness and detail.

To reconstruct the estimated image while preserving the sharp edges, total variation (TV) regularization is introduced in [19]. TV regularization is one of the most successful techniques for reconstructing images. In the field of denoising, various noise types are effectively removed using this regularization strategy [20]. Unlike Tikhonov regularization, TV regularization is employed in the image restoration process under the assumption that the image gradient distribution has a Laplace distribution. Therefore, high-frequency information, such as edge information or details, can be effectively restored. TV regularization is defined as follows:

(11) R(x)=||∇x||,

where *∇* denotes the first derivative linear operator. TV regularization is isotropic if the norm ||·|| denotes the $L_{2}$-norm, and it is anisotropic if it denotes the $L_{1}$-norm.

Although TV regularization exhibits excellent performance in various image processing fields, recent works have focused on presenting constraints with consideration of the image characteristics. In research on the properties of natural images [21,22], the statistic of real-world scenes substantially follows a heavy-tailed distribution in their gradients, which is modeled as a hyper-Laplacian prior ($p\left(x\right)\propto \exp(-k||\nabla x|{|}_{p}^{p})$). This prior is incorporated as $L_{p}$ regularization, and it is defined as follows:

(12) $$R\left(x\right)={\left||\nabla x|\right|}_{p}^{p},$$

where the norm $\left|\right|\cdot {\left|\right|}_{p}^{p}$ denotes the $L_{p}$-norm and the value of p is typically set to [0.5,0.8].

### 3.2. Constraints

**Figure 5:**
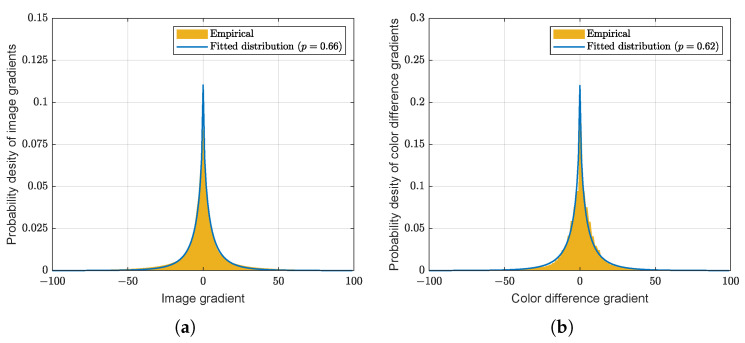
Probability distributions from Kodak and McMaster datasets: (**a**) Distribution of image gradients and (**b**) distribution of color difference gradients. The empirical distributions of image gradients and color difference gradients follow the probability distributions of p=0.66 and 0.62, respectively.

In the spirit of a hyper-Laplacian prior, the proposed constraint consists of spatial regularization to encourage high frequencies within each color channel. It additionally fosters intercolor regularization to enforce the homogeneity of the edges or details in the color channels. The first constraint $R_{1}\left(x\right)$ coerces sharp edges in the estimated image. If the prior knowledge of the image gradient is considered, an optimal solution can be found. Figure 5a presents the probability density of the first-order derivative using 42 images from the Kodak and McMaster datasets [23]. The empirical distribution can be modeled as a hyper-Laplacian distribution with p=0.66. This property allows a regularization term to employ a hyper-Laplacian prior in the deconvolution problem. Therefore, $R_{1}\left(x\right)$ is represented as follows:

(13) $$R_{1}\left(x\right)={\left||\nabla x|\right|}_{p}^{p},$$

where ∇ denotes the first derivative operator and p=0.66.

The second constraint $R_{2}\left(x\right)$ is chosen to encourage smoothness in the differences between the color components. In most demosaicing, color interpolation is performed under the assumption of a high correlation among the three color bands [4,5,6,7,8]. This property is also valid for natural color images. The optimal $L_{p}$-norm can be selected through the probability density of the color difference gradient, as presented in Figure 5b. Similar to the case of $R_{1}\left(x\right)$, a hyper-Laplacian distribution with p=0.62 allows the regularization term to deploy $L_{p}$-norm prior in the image restoration process. The term $R_{2}\left(x\right)$ is expressed as follows:

(14) $$\begin{array}{cc} R_{2}\left(x\right)= & ||\nabla \left(+2x_{R}-x_{G}-x_{B}\right){\left|\right|}_{p}^{p}\\ + & ||\nabla \left(-x_{R}+2x_{G}-x_{B}\right){\left|\right|}_{p}^{p}\\ + & ||\nabla \left(-x_{R}-x_{G}+2x_{B}\right){\left|\right|}_{p}^{p}. \end{array}$$

This regularization term can be expressed in the form $R_{2}\left(x\right)={\left||\nabla Mx|\right|}_{p}^{p}$, where

(15) $$M=\left(\begin{array}{ccc} \begin{array}{ccc} 2 & 0 & \cdots \\ 0 & 2 & \\ \vdots & \; & \ddots \end{array} & \begin{array}{ccc} -1 & 0 & \cdots \\ 0 & -1 & \\ \vdots & \; & \ddots \end{array} & \begin{array}{ccc} -1 & 0 & \cdots \\ 0 & -1 & \\ \vdots & \; & \ddots \end{array}\\ \begin{array}{ccc} -1 & 0 & \cdots \\ 0 & -1 & \\ \vdots & \; & \ddots \end{array} & \begin{array}{ccc} 2 & 0 & \cdots \\ 0 & 2 & \\ \vdots & \; & \ddots \end{array} & \begin{array}{ccc} -1 & 0 & \cdots \\ 0 & -1 & \\ \vdots & \; & \ddots \end{array}\\ \begin{array}{ccc} -1 & 0 & \cdots \\ 0 & -1 & \\ \vdots & \; & \ddots \end{array} & \begin{array}{ccc} -1 & 0 & \cdots \\ 0 & -1 & \\ \vdots & \; & \ddots \end{array} & \begin{array}{ccc} 2 & 0 & \cdots \\ 0 & 2 & \\ \vdots & \; & \ddots \end{array} \end{array}\right),$$

and M is an 3MN×3MN dimensional matrix.

Each regularization term of $R_{1}\left(x\right)$ and $R_{2}\left(x\right)$ is incorporated into the objective function of (8), and the overall objective function is represented as follows:

(16) $$\hat{x}=\arg\min_{x}\left\{||y-{DHx||}_{2}^{2}+\lambda_{1}{\left||\nabla x|\right|}_{p}^{p}+\lambda_{2}{\left||\nabla Mx|\right|}_{p}^{p}\right\},$$

where $\lambda_{1}$ and $\lambda_{2}$ are regularization parameters for the constraints of color components and color difference components, respectively. As $R_{1}\left(x\right)$ and $R_{2}\left(x\right)$ have similar *p* values near 2/3, we assume p=2/3 in the following optimization problem for convenience.

### 3.3. Optimization

In this subsection, we describe how to optimize the objective function of Equation (16). The minimization of an objective function with $L_{p}$ regularization engenders difficulty in obtaining closed-form solutions because of the non-differentiability and non-linearity of the $L_{p}$ regularization. To efficiently recover estimated image x and solve this problem, a strategy based on alternating minimization [24], known as half-quadratic splitting, is employed. Two auxiliary variables w and v are introduced to approximate ∇x and ∇Mx among the non-differentiable terms $\left|\right|\cdot {\left|\right|}_{p}^{p}$, respectively. The approximation model to Equation (16) can be expressed as follows:

(17) $$\begin{array}{cc} \hat{x}=\arg\min_{x,w,v} & \{||z-{Hx||}_{2}^{2}+\theta_{1}||\nabla x-{w||}_{2}^{2}\\ \; & +\lambda_{1}{\left||w|\right|}_{p}^{p}+\theta_{2}||\nabla Mx-{v||}_{2}^{2}+\lambda_{2}{\left||v|\right|}_{p}^{p}\}, \end{array}$$

where w and v are the auxiliary variables, $\theta_{i}$ denotes the auxiliary regularization parameter, and z represents the estimated full-color image. Alternating minimization guarantees that the minimizer of Equation (17) converges to that of Equation (16) as the value of $\theta_{i}$ moves toward *∞*. Therefore, the objective function in Equation (17) is convex and differentiable. This formulation allows the minimization problem with respect to the others to obtain a closed-form formula if one of the three variables, w, v, and x, is fixed.

Note that vector z is first estimated based on the demosaicing method that satisfies the condition y=Dz. The choice of the demosaicing method is not relevant; however, the state-of-the-art demosaicing method (e.g., ARI [8]) is advantageous in reducing color artifacts. Although the effects of subsampling cannot be completely eliminated, this process enables the objective function to negate the subsampling operator of matrix D.

Since w and v are separable in a given x, the minimizer of the objective function is readily obtained. By fixing x, the formulation of Equation (17) is separable in w and v. Based on the alternative minimization method [24], the minimization problem can be transformed into two subproblems, as follows:

(18) $$\begin{array}{cc} \hat{w} & =\arg\min_{w}\left\{||w-{\nabla x||}_{2}^{2}+\frac{\lambda_{1}}{\theta_{1}}{\left||w|\right|}_{p}^{p}\right\}, \end{array}$$

(19) $$\begin{array}{cc} \hat{v} & =\arg\min_{v}\left\{||v-{\nabla Mx||}_{2}^{2}+\frac{\lambda_{2}}{\theta_{2}}{\left||v|\right|}_{p}^{p}\right\}. \end{array}$$

Each objective function in (18) and (19) is a single-variable problem. Although it is difficult to derive an exact solution for these objective functions, the minimization can be obtained using a numerical root-finder, such as the Newton–Raphson method. In the special case of p=2/3, the exact analytic solution for non-zero w and v is described in [21]. Specifically, the multidimensional minimizer of ${\alpha ||s||}_{p}^{p}+||s-{t||}_{2}^{2}$ is extended to the scalar minimizer of ${\alpha |s|}^{p}+{\left|s-t\right|}^{2}$. By calculating the derivative of ${\alpha |s|}^{p}+{\left|s-t\right|}^{2}$ with respect to *s*, the minimizer is given by:

(20) $${\alpha p|s|}^{p-1}+2\left|s-t\right|=0.$$

For p=2/3, the equation can be expressed as follows:

(21) $$\begin{array}{cc} {\alpha |s|}^{-1/3}+3\left|s-t\right| & =0,\\ \alpha^{3}{\left|s\right|}^{-1}+27{\left|s-t\right|}^{3} & =0,\\ s^{4}-3ts^{3}+3t^{2}s^{2}-t^{3}s+\frac{\alpha^{3}}{27} & =0. \end{array}$$

Therefore, the solution of each objective function in (18) and (19) can be solved numerically through the root of the quartic polynomial.

Note that if p=1, then the objective functions in (18) and (19) will be equal to the TV regularized problem. Although the proposed method has an optimal solution with approximately p=2/3 and a corresponding solution is derived above, the TV regularization remains a useful strategy in terms of speed and implementation. Each solution of (18) and (19) with p=1 is given by the one-dimensional shrinkage, as follows:

(22) $$\begin{array}{cc} \hat{w} & =\frac{\nabla x}{{\left||\nabla x|\right|}_{1}}{\max(||\nabla x||}_{1}-\frac{\lambda_{1}}{2\theta_{1}},0), \end{array}$$

(23) $$\begin{array}{cc} \hat{v} & =\frac{\nabla Mx}{{\left||\nabla Mx|\right|}_{1}}{\max(||\nabla Mx||}_{1}-\frac{\lambda_{2}}{2\theta_{2}},0). \end{array}$$

The solution to this problem can be achieved component-wise.

For fixed w and v variables, the objective function in (17) can be simplified as a least-squares formulation as

(24) $$\begin{array}{c} \hat{x}=\arg\min_{x}\left\{||z-{Hx||}_{2}^{2}+\theta_{1}||\nabla x-{w||}_{2}^{2}+\theta_{2}||\nabla Mx-{v||}_{2}^{2}\right\}. \end{array}$$

Moreover, the objective function is convex and differentiable with respect to x. As the objective function is composed of quadratic terms, the corresponding optimal solution of Equation (24) becomes a least-squares problem as follows:

(25) $$\begin{array}{cc} (H^{T}H+\theta_{1}\nabla^{T}\nabla & +\theta_{2}{\left(\nabla M\right)}^{T}\left(\nabla M\right))x=H^{T}z+\theta_{1}\nabla^{T}w+\theta_{2}{\left(\nabla M\right)}^{T}v. \end{array}$$

The solution can be obtained by solving its normal equations.

We summarize the proposed crosstalk correction process based on multi-channel deconvolution in Algorithm 1 with the continuation technique on regularization parameter $\theta_{i}$. The continuous scheme is widely used with the penalty method to speed up the overall convergence. As a small value of $\theta_{i}$ is initially set and gradually increases in iterations, the convergence speed can be accelerated. As the value of $\theta_{i}$ moves toward inf, the minimizer of Equation (17) transformed by the quadratic penalty method converges to that of Equation (16). In terms of convergence, the objective function in Equation (17) is convex in x, w and v.
**Algorithm 1** Crosstalk Correction based on $L_{p}$-regularized Multi-channel Deconvolution.**Input:** The observed image y, the subsamling matrix D, the crosstalk matrix H, and the regularization parameters $\lambda_{i}$**Output:** The reconstructed image $\hat{x}$  *Initialization*: $x_{0}=0$  Solve z based on the demosaicing method (y=Dz)  $\theta_{i}\leftarrow \lambda_{i}$1:**repeat**2:   Solve w according to Equation (18)3:   Solve v according to Equation (19)4:   Solve x according to Equation (24)5:   $\theta_{i}\leftarrow 2\theta_{i}$6:**until** $\theta_{i}<\theta^{max}$7:**return** $\hat{x}$

In implementation terms, the computation of each block in Equation (25) can be accelerated by a two-dimensional (2D) fast Fourier transform (FFT) at each iteration. Under the periodic boundary condition for x, $\nabla^{T}\nabla$, and all block matrices in $H^{T}H$ are block circulants. Specifically, the matrix on the left-hand side of Equation (25) can be precomputed once before the iterations. At each iteration, six FFTs are applied to w and v. Then, three inverse FFTs are computed to obtain x. Therefore, a total of nine FFTs (including three inverse FFTs) are required to solve Equation (25).

## 4. Experimental Results

The performance of the proposed method was evaluated using the color peak signal-to-noise ratio (CPSNR) and structural similarity index (SSIM) [25] as objective image quality metrics. The former was used to evaluate the intensity differences between the original image and the estimated image; the latter was employed to evaluate the structural similarity in terms of the human visual system. In particular, SSIM is suitable for comprehensively determining the similarity between luminance and chrominance components.

### 4.1. Datasets

Several experiments were conducted to verify the performance of the proposed method using artificially degraded images. For the comparisons, we generated crosstalk degradation on four public benchmark datasets: Kodak, McMaster [23], Set5 [26], and Set14 [27]. The point spread function of crosstalk is inspired by the crosstalk kernel given by [14]. Moreover, two levels of degradation were tested with consideration of the crosstalk phenomenon that intensifies in accordance with the pixel size. For *crosstalk degradation 1*, the synthetic datasets were generated by convolving the ground truth (GT) images within the given dynamic range [0,1] and the crosstalk kernels of the 2D Gaussian kernel with a standard deviation of $\sigma_{g}=0.45$. They were also generated by adding Gaussian noise with a standard deviation of $\sigma_{n}=0.01$. For *crosstalk degradation 2*, the Gaussian crosstalk kernel with a standard deviation of $\sigma_{g}=0.60$ and a Gaussian noise with a standard deviation of $\sigma_{n}=0.04$ were used. We assume that the crosstalk kernel is space-invariant.

### 4.2. Compared Methods

For performance comparisons, we implemented four conventional methods. The first conventional method (CM1) was explained as the ARI method in [8]. It only performed demosaicing and ignored the crosstalk kernel. The second (CM2), described as Lucy-demosaicing in [16], performed single-channel deconvolution based on the Lucy–Richardson method [28]. The third (CM3) was inspired by CM2 and applied TV-based single-channel deconvolution [24]. For CM2 and CM3, deconvolution was performed in the subsampled domain, followed by demosaicing. The fourth method (CM4) was deemed a color correction method in [13]. It focuses on color correction with consideration of the crosstalk kernel. For CM4, the CFA image was first demosaiced by the various demosaicing methods. The fifth (CM5), sixth (CM6), and seventh (CM7) performed single-channel deconvolution based on deep-learning approach [29,30,31]. Each method uses a convolution neural network (CNN) to solve a constraint optimization problem. In a similar way to CM3 and CM4, deconvolution performed in the subsampled domain for CM5, CM6, and CM7 using pre-trained models. In this experiment, ARI demosaicing [8] was applied equally to various conventional methods requiring demosaicing. The parameters were chosen to achieve the highest CPSNR values for all test images.

### 4.3. Comparisons

The quantitative evaluation of the Kodak and McMaster datasets [23] for *crosstalk degradation 1* in terms of the CPSNR and SSIM values is demonstrated in Table 1. It is observed that the proposed method improved the performance. It achieved the highest CPSNR and SSIM values among all methods, including demosaicing (CM1), deconvolution (CM2 and CM3), color correction (CM4), and deep-learning-based deconvolution approach (CM5, CM6, and CM7). Note that the higher the CPSNR value is, the closer the estimated image is to the original image. Moreover, the higher the SSIM value, the better the perceived quality. The widening performance gap between the conventional methods and the proposed method can be confirmed for *crosstalk degradation 2* in Table 2. In other words, the proposed method is robust against both crosstalk degradation and noise. Quantitative assessments for the Set5 [26] and Set14 [27], including the Kodak and McMaster datasets [23], are summarized in Table 3.

**Figure 6:**
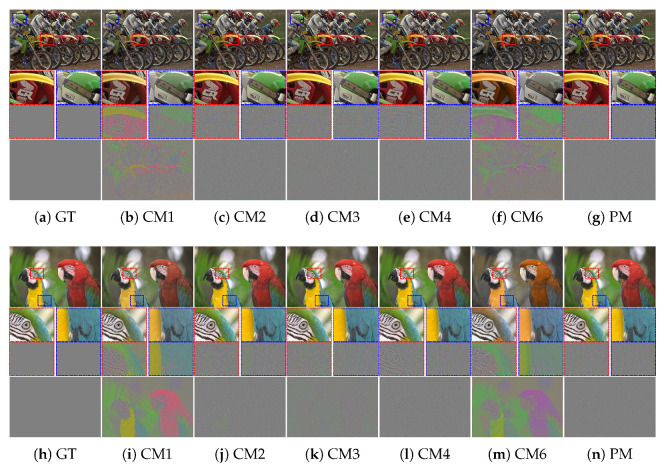
Visual comparison of restored images, enlarged parts, and difference maps from *crosstalk degradation 1* (crosstalk kernel with $\sigma_{g}=0.45$ and noise with $\sigma_{n}=0.01$): (**a**) Ground truth, (**b**) CM1, (**c**) CM2, (**d**) CM3, (**e**) CM4, (**f**) CM6, and (**g**) PM. (**h**–**n**) Same methods as in (**a**–**g**). The first and second rows present the results of *Bike* and *Parrot* in the Kodak dataset, respectively.

A comparison of the qualitative evaluation for crosstalk correction is visualized in Figure 6. In CM1, where only demosaicing was applied, color degradation is clearly observed. When the degree of crosstalk degradation is insignificant, it is defined as *crosstalk degradation 1*. The single-channel deconvolution methods (CM2 and CM3) show relatively good results in terms of improving color fidelity and producing sharp details. However, noise amplification is not completely considered. In CM4, which focused on color correction, blurring artifacts are not at all improved. The deep-learning-based deconvolution methods (CM5, CM6, and CM7) show usable results in terms of reducing noise and producing sharp details. However, the color fidelity has improved in a limited way. In contrast, the proposed method based on multi-channel deconvolution successfully reconstructed edge sharpness and overcame color degradation without noise amplification.

**Figure 7:**
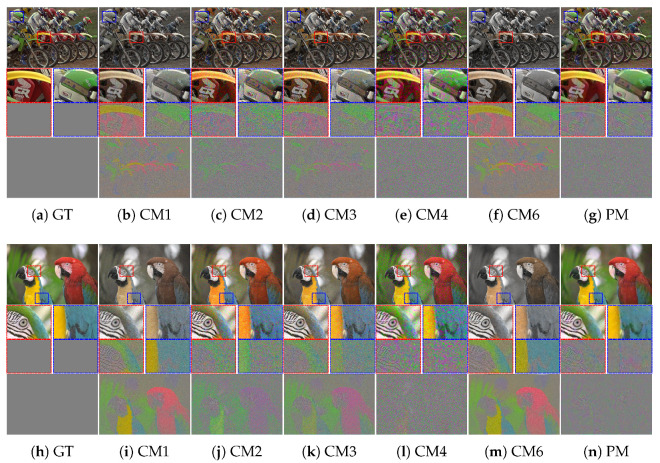
Visual comparison of restored images, enlarged parts, and difference maps from *crosstalk degradation 2* (crosstalk kernel with $\sigma_{g}=0.6$ and noise with $\sigma_{n}=0.04$): (**a**) Ground truth, (**b**) CM1, (**c**) CM2, (**d**) CM3, (**e**) CM4, (**f**) CM6, and (**g**) PM. (**h**–**n**) Same methods as in (**a**–**g**). The first and second rows present the results of *Bike* and *Parrot* in the Kodak dataset, respectively.

**Figure 8:**
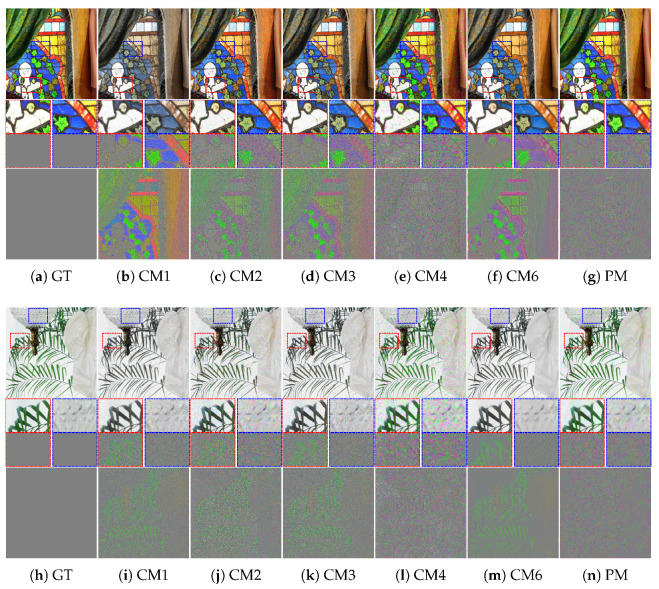
Visual comparison of restored images, enlarged parts, and difference maps from *crosstalk degradation 2* (crosstalk kernel with $\sigma_{g}=0.6$ and noise with $\sigma_{n}=0.04$): (**a**) Ground truth, (**b**) CM1, (**c**) CM2, (**d**) CM3, (**e**) CM4, (**f**) CM6, and (**g**) PM. (**h**–**n**) Same methods as in (**a**–**g**). The first and second rows present the results of *mcm1* and *mcm4* in the McMaster dataset, respectively.

Furthermore, as the degree of crosstalk degradation increased, the performance difference between the conventional methods and the proposed method became more apparent. Figure 7 and Figure 8 present the results for *crosstalk degradation 2*. CM1 shows noisy results without overcoming color degradation and blurring artifacts. The majority of single-channel deconvolution methods (CM2 and CM3) exhibit limitations in terms of color fidelity because deconvolution was performed in the subsampled domain. With respect to noise, CM3 shows slightly better results than CM2. In CM4, the color fidelity is improved close to the ground truth; however, the noise is amplified, appearing like ink stains in the pixels with noise. However, as is clearly observed, the proposed method based on multi-channel deconvolution improves the color fidelity and produces much sharper details than the other methods. Specifically, it produces the fewest artifacts, such as noise amplification. In addition, the visual comparison of the difference image shows that the proposed method achieves a low error rate. Therefore, the proposed method yields more natural and credible results than the conventional methods.

### 4.4. Influence of Parameters

**Figure 9:**
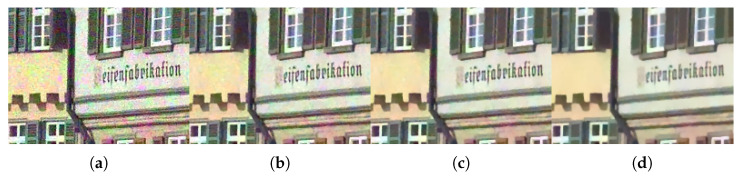
Influence of regularization parameter $\lambda_{1}$ for test image *House* for *crosstalk degradation 2* (crosstalk kernel with $\sigma_{g}=0.6$ and noise with $\sigma_{n}=0.04$): (**a**) $\lambda_{1}=0.01$, (**b**) $\lambda_{1}=0.03$, (**c**) $\lambda_{1}=0.05$, and (**d**) $\lambda_{1}=0.1$.

**Figure 10:**
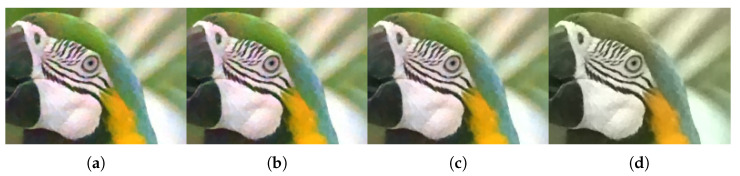
Influence of regularization parameter $\lambda_{2}$ for test image *Parrot* for *crosstalk degradation 2* (crosstalk kernel with $\sigma_{g}=0.6$ and noise with $\sigma_{n}=0.04$): (**a**) $\lambda_{2}=0.0001$, (**b**) $\lambda_{2}=0.001$, (**c**) $\lambda_{2}=0.01$, and (**d**) $\lambda_{2}=0.1$.

The proposed method involves regularization parameters $\lambda_{i}$, which manage the trade-off between the fidelity to the data and smoothness of the solution. To analyze the effects of the values of $\lambda_{i}$ on the crosstalk correction method, we performed experiments with different parameter settings. The value of $\lambda_{i}$ should be determined empirically based on experiments so that the restored images have reasonable signal-to-noise ratios. Figure 9 and Figure 10 present examples of the influence of $\lambda_{1}$ and $\lambda_{2}$ in the case of *crosstalk degradation 2*. The results with small values of $\lambda_{1}$ are much sharper and noisy, whereas the results with large values of $\lambda_{1}$ are seemingly smoothed and have less noise. Smaller values of $\lambda_{2}$ tend to yield more vivid color, whereas large values of $\lambda_{2}$ may be desaturated and have fewer color artifacts.

**Figure 11:**
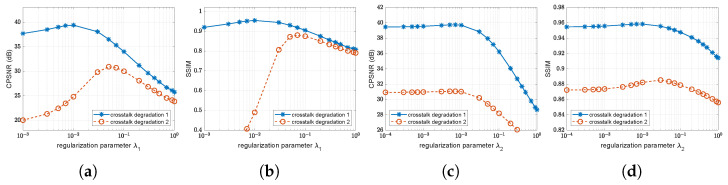
Objective performance comparison for test image *Parrot* according to regularization parameters $\lambda_{1}$ and $\lambda_{2}$: (**a**) CPSNR values versus values of $\lambda_{1}$; (**b**) SSIM values versus values of $\lambda_{1}$; (**c**) CPSNR values versus values of $\lambda_{2}$; and (**d**) SSIM values versus values of $\lambda_{2}$.

Figure 11 demonstrates that crosstalk correction can be effectively performed in a range of $\lambda_{1}$, that is, within [0.005,0.05] and [0.05,0.01] for *crosstalk degradation 1 and 2*, respectively. It is appropriate to select the values of $\lambda_{2}$ within [0.0003,0.003]. Therefore, choosing the appropriate $\lambda_{i}$ according to the level of degradation will improve the subjective and objective performances.

### 4.5. Convergence Analysis

**Figure 12:**
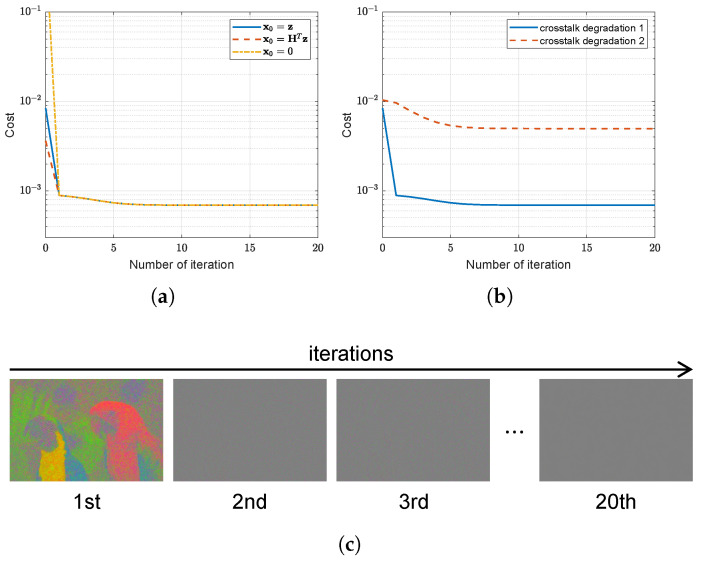
Convergence for test image *Parrot*: (**a**) Value of cost function versus iteration number of various initial conditions for *crosstalk degradation 1* (crosstalk kernel with $\sigma_{g}=0.45$ and noise with $\sigma_{n}=0.01$); (**b**) value of cost function versus iteration number of various degradation conditions; and (**c**) visualization of residual image versus iteration number at *crosstalk degradation 2* (crosstalk kernel with $\sigma_{g}=0.6$ and noise with $\sigma_{n}=0.04$).

Finally, we analyzed the convergence behavior of multi-channel deconvolution for crosstalk correction. The criterion $\left|\right|x_{n+1}-x_{n}{\left|\right|}^{2}/\left|\right|x_{n}{\left|\right|}^{2}<\eta$ was used to terminate the iteration, where η was set to $10^{-4}$. The propose method converges for various initial conditions, as plotted in Figure 12a. For the initial estimates, $x_{0}$, a blank image ($x_{0}=0$), a demosaiced version of the observed image ($x_{0}=z$), or a degraded version of the demosaiced image ($x_{0}=H^{T}z$) was employed. For the experiment on various levels of crosstalk degradation, the objective function decreased in several iterations, as illustrated in Figure 12b. In addition, the visualization in Figure 12c shows that the residual image converges to the image with no value as the iteration progresses. Therefore, the objective function with a hyper-Laplacian prior converged in several iterations for some variables through the efficient solver.

## 5. Discussion

The crosstalk problem generated by the crosstalk kernel in the subsampled domain causes not only spatial degradation, but also spectral degradation at the same time. Single-channel deconvolution does not consider the characteristics of CFA, and as a result, there is a limit to solving complex problems. In this study, it is shown that the crosstalk problem can be defined as a multi-channel degradation model. In addition, a method to solve the defined modeling is presented, and an effective objective function and optimization method are proposed. As can be seen from the experimental results, single-channel deconvolution can solve the blurring, but cannot solve the desaturation regardless of various methods. The color correction method is able to improve color fidelity, but the problems regarding blur and noise still remained. On the other hand, the proposed method is able to improve both spatial resolution and color fidelity without noise amplification, and it has been demonstrated that a synergistic effect with the state-of-the-art demosaicing algorithm can be achieved.

One of the important points is that the regularization parameter in the proposed method controls the smoothness. Although we have suggested the effective range of regularization parameters empirically, they will be important variables affecting the results. Overall, the main concept of the proposed multi-channel deconvolution for crosstalk correction is to overcome spatial and spectral degradation simultaneously, and its validity has been proven. The proposed multi-channel deconvolution approach can provide new insights into solving the crosstalk problem that occurs in the image acquisition system.

## 6. Conclusions

A crosstalk correction method based on $L_{p}$-regularized multi-channel deconvolution was herein presented. The crosstalk problem with complex spatial and spectral degradation is formulated as a multi-channel degradation model, and an objective function with a hyper-Laplacian prior is designed. The proposed method based on multi-channel deconvolution improves color fidelity and produces sharp edges without obvious artifacts, such as noise amplification. Furthermore, an efficient solver minimizes the objective function for crosstalk correction. The cost function gradually converges toward a minimum value for certain variables. The proposed method was compared with conventional crosstalk correction methods using synthetic datasets according to various crosstalk and noise levels. Experimental results demonstrated that the proposed method outperformed the conventional methods in terms of the CPSNR and SSIM values. However, in terms of speed, the weakness of the proposed method is that the computational complexity is high to perform real-time processing in camera imaging system. In further research, we will implement the proposed method with multiple GPUs for a real-time performance. We believe that the proposed method will be applied to the application of imaging systems in many fields by overcoming the physical limitations of the image sensor.

## Figures and Tables

**Table 1:** Objective Performance Comparison of the Kodak 24 and McMaster 18 Images in CPSNR (dB) and SSIM for *Crosstalk Degradation 1* (crosstalk kernel with $\sigma_{g}=0.45$ and noise with $\sigma_{n}=0.01$).

Kodak dataset																
	CPSNR								SSIM							
No.	CM1	CM2	CM3	CM4	CM5	CM6	CM7	PM	CM1	CM2	CM3	CM4	CM5	CM6	CM7	PM
1	30.59	35.15	35.20	33.72	26.47	29.56	24.51	35.67	0.9527	0.9553	0.9568	0.9494	0.8497	0.9483	0.9223	0.9628
2	23.89	34.98	35.44	35.91	29.28	23.97	24.45	36.85	0.8992	0.8887	0.8918	0.9068	0.8428	0.7832	0.8429	0.9278
3	27.47	36.63	36.94	37.13	27.75	25.19	25.22	38.71	0.9165	0.9034	0.9120	0.9174	0.8731	0.8991	0.8797	0.9533
4	27.17	35.45	35.75	36.43	25.82	24.26	23.02	37.15	0.9259	0.9112	0.9153	0.9265	0.8355	0.8653	0.8745	0.9411
5	30.13	35.02	35.11	33.68	27.34	28.36	25.12	35.42	0.9549	0.9585	0.9603	0.9581	0.8937	0.9467	0.9283	0.9679
6	30.37	35.49	35.70	35.03	25.62	27.85	24.72	36.39	0.9484	0.9374	0.9413	0.9425	0.8444	0.9446	0.9114	0.9572
7	30.76	36.66	36.88	37.11	27.16	28.59	25.33	38.72	0.9417	0.9247	0.9306	0.9373	0.9226	0.9292	0.9073	0.9658
8	30.55	33.21	33.38	31.61	27.58	30.73	24.19	33.61	0.9543	0.9520	0.9550	0.9507	0.8951	0.9585	0.9257	0.9586
9	33.20	36.19	36.47	36.32	31.01	30.14	24.78	38.01	0.9318	0.8872	0.9028	0.9106	0.9180	0.9364	0.8796	0.9442
10	34.33	36.40	36.53	36.44	29.09	29.64	24.14	37.90	0.9379	0.9093	0.9177	0.9262	0.9140	0.9360	0.8880	0.9480
11	31.58	35.37	35.65	35.20	26.62	29.04	25.20	36.44	0.9406	0.9255	0.9320	0.9323	0.8241	0.9442	0.9014	0.9482
12	30.71	36.86	36.72	37.29	24.46	27.02	24.74	38.60	0.9345	0.9080	0.9133	0.9238	0.8388	0.9188	0.8824	0.9459
13	29.33	33.38	33.41	31.45	25.82	28.22	24.66	33.95	0.9481	0.9585	0.9598	0.9473	0.8135	0.9513	0.9278	0.9646
14	27.92	34.45	34.71	34.39	27.80	27.12	24.97	35.20	0.9391	0.9400	0.9441	0.9443	0.8538	0.9361	0.9085	0.9518
15	28.21	34.87	35.20	35.60	26.08	25.00	24.27	36.81	0.9194	0.8913	0.8968	0.9119	0.8485	0.8789	0.8699	0.9381
16	34.48	36.58	36.93	36.94	28.61	32.19	25.08	37.98	0.9435	0.9234	0.9307	0.9334	0.8822	0.9481	0.9037	0.9531
17	34.86	36.16	36.54	36.21	30.75	32.62	23.20	37.63	0.9419	0.9186	0.9286	0.9334	0.9025	0.9461	0.8905	0.9539
18	30.18	34.31	34.45	33.62	27.99	29.04	23.44	34.78	0.9348	0.9292	0.9355	0.9338	0.8450	0.9268	0.8977	0.9434
19	31.80	35.65	35.83	34.88	28.37	30.25	23.02	36.44	0.9382	0.9238	0.9306	0.9319	0.8526	0.9370	0.8905	0.9476
20	32.17	36.63	36.78	36.56	27.79	31.10	21.78	38.19	0.9367	0.9153	0.9218	0.9278	0.8861	0.9365	0.8730	0.9521
21	31.98	35.41	35.59	34.82	28.57	32.12	24.12	36.44	0.9382	0.9074	0.9133	0.9215	0.8921	0.9296	0.8926	0.9492
22	29.95	34.95	35.09	35.11	26.10	28.13	24.60	35.92	0.9305	0.9164	0.9229	0.9275	0.8106	0.9215	0.8946	0.9399
23	25.74	36.88	36.90	37.38	26.76	23.06	24.95	39.36	0.9135	0.8965	0.9044	0.9153	0.8902	0.8738	0.8706	0.9522
24	31.34	33.71	33.79	32.90	28.13	29.77	23.41	34.30	0.9488	0.9411	0.9451	0.9440	0.8937	0.9525	0.9177	0.9592
Avg.	30.36	35.43	35.63	35.24	27.54	28.46	24.29	36.69	0.9363	0.9218	0.9276	0.9314	0.8676	0.9229	0.8950	0.9511
McMaster dataset																
	CPSNR								SSIM							
No.	CM1	CM2	CM3	CM4	CM5	CM6	CM7	PM	CM1	CM2	CM3	CM4	CM5	CM6	CM7	PM
1	22.33	29.21	29.18	28.96	26.31	20.12	20.85	29.12	0.8460	0.8968	0.8967	0.8920	0.8195	0.8017	0.8329	0.8992
2	27.01	33.42	33.37	33.35	27.43	25.36	22.16	33.88	0.8754	0.8987	0.8994	0.9026	0.7900	0.8190	0.8291	0.9196
3	28.40	33.07	33.09	32.36	27.72	25.73	22.17	33.41	0.9245	0.9348	0.9378	0.9377	0.8910	0.9025	0.8764	0.9549
4	30.85	34.71	34.90	34.64	30.34	27.17	20.35	36.21	0.9516	0.9372	0.9425	0.9474	0.9504	0.9583	0.8993	0.9713
5	27.55	33.53	33.53	33.53	27.53	26.61	22.19	33.88	0.9077	0.9188	0.9208	0.9243	0.8568	0.8773	0.8752	0.9370
6	28.08	35.77	35.71	35.93	28.83	27.25	23.31	36.50	0.9045	0.9263	0.9279	0.9320	0.8500	0.8650	0.8806	0.9447
7	30.03	35.72	35.97	35.52	30.09	28.78	22.93	36.11	0.9254	0.9313	0.9342	0.9331	0.8348	0.9079	0.8610	0.9458
8	33.80	35.85	35.98	35.36	32.21	31.44	22.27	36.77	0.9132	0.9104	0.9146	0.9122	0.8891	0.9021	0.7697	0.9449
9	26.06	34.77	34.73	34.87	28.09	24.89	21.68	35.80	0.8841	0.9027	0.9036	0.9132	0.8431	0.8100	0.8424	0.9409
10	24.01	35.74	35.58	35.87	27.79	22.06	22.55	36.66	0.8582	0.9185	0.9181	0.9218	0.8137	0.7644	0.8305	0.9464
11	24.45	36.28	36.06	36.43	27.14	22.83	22.34	37.36	0.8150	0.9118	0.9075	0.9161	0.7336	0.7052	0.7810	0.9426
12	25.63	36.12	36.09	36.03	30.77	27.27	22.36	37.69	0.8938	0.8973	0.8988	0.9094	0.8624	0.8367	0.8471	0.9455
13	27.79	36.16	36.19	36.88	33.04	31.32	22.60	38.79	0.9015	0.8639	0.8675	0.8895	0.8838	0.8592	0.8435	0.9323
14	26.26	35.66	35.55	36.04	27.51	25.07	22.36	37.04	0.8740	0.8906	0.8924	0.9007	0.8084	0.8005	0.8248	0.9296
15	24.16	35.85	35.71	36.04	28.61	22.12	22.41	37.13	0.8509	0.8904	0.8902	0.8971	0.7458	0.7069	0.7838	0.9280
16	21.74	33.66	33.48	33.11	27.96	18.23	21.67	33.17	0.8098	0.9346	0.9332	0.9326	0.8141	0.7283	0.8265	0.9369
17	22.42	32.78	32.65	32.71	26.90	19.07	21.59	32.92	0.8137	0.9218	0.9202	0.9239	0.7896	0.6346	0.8147	0.9326
18	25.46	33.99	33.91	34.00	27.56	22.81	21.54	34.70	0.9103	0.9248	0.9257	0.9293	0.8581	0.8628	0.8593	0.9419
Avg.	26.45	34.57	34.54	34.53	28.66	24.90	22.07	35.40	0.8811	0.9117	0.9128	0.9175	0.8352	0.8190	0.8377	0.9386

**Table 2:** Objective Performance Comparison of the Kodak 24 and McMaster 18 Images in CPSNR (dB) and SSIM for *Crosstalk Degradation 2* (crosstalk kernel with $\sigma_{g}=0.6$ and noise with $\sigma_{n}=0.04$).

Kodak dataset																
	CPSNR								SSIM							
No.	CM1	CM2	CM3	CM4	CM5	CM6	CM7	PM	CM1	CM2	CM3	CM4	CM5	CM6	CM7	PM
1	23.45	20.40	22.96	20.14	22.80	23.14	19.54	25.91	0.7248	0.4425	0.6344	0.4994	0.6268	0.7946	0.4642	0.7379
2	17.06	21.14	19.98	18.22	21.64	16.51	18.54	29.96	0.5310	0.2356	0.2662	0.1925	0.3376	0.5778	0.2390	0.7700
3	20.82	19.61	22.39	17.56	23.37	20.41	19.30	30.91	0.5760	0.1969	0.3985	0.1917	0.6379	0.7723	0.2364	0.8465
4	20.61	20.16	21.66	18.58	21.33	19.68	18.52	29.88	0.6019	0.2448	0.4159	0.2518	0.4217	0.7373	0.2706	0.7980
5	23.23	21.09	23.01	20.60	23.57	23.24	20.36	25.96	0.7426	0.5307	0.6606	0.5354	0.7330	0.8275	0.5539	0.8009
6	23.54	19.94	22.87	19.06	23.15	23.17	18.89	27.07	0.6732	0.3232	0.5541	0.3599	0.6241	0.8006	0.3622	0.7546
7	23.59	20.26	23.50	18.49	24.08	23.28	19.91	29.91	0.6542	0.3197	0.5078	0.3259	0.7194	0.8383	0.3575	0.8836
8	23.79	20.63	23.20	20.05	24.93	24.58	19.79	24.89	0.7587	0.5414	0.6901	0.5629	0.7924	0.8487	0.5709	0.7847
9	25.66	19.83	24.46	17.21	28.08	25.80	19.55	30.53	0.6207	0.2349	0.4672	0.2356	0.8367	0.8285	0.2810	0.8570
10	26.63	19.85	24.70	18.11	27.69	26.75	19.46	30.25	0.6299	0.2316	0.4837	0.2492	0.8225	0.8265	0.2868	0.8423
11	24.58	20.34	23.75	18.96	24.47	24.81	19.91	28.09	0.6435	0.3045	0.5264	0.3133	0.7058	0.7934	0.3507	0.7661
12	23.96	19.53	23.16	17.63	23.00	23.38	19.12	30.74	0.5995	0.1822	0.4279	0.1920	0.5484	0.7995	0.2405	0.8114
13	22.82	20.23	22.58	20.42	20.91	22.93	19.41	24.17	0.7306	0.4803	0.6695	0.5378	0.5656	0.7828	0.5030	0.7413
14	21.08	20.48	22.78	20.13	23.55	21.12	19.85	27.17	0.6743	0.3728	0.5626	0.4058	0.6492	0.7627	0.4063	0.7613
15	21.82	20.72	22.46	18.61	23.18	21.37	18.69	30.04	0.5925	0.2547	0.4325	0.2310	0.6755	0.7784	0.2741	0.8176
16	26.41	19.88	24.56	18.09	26.03	27.01	19.74	29.20	0.6289	0.2136	0.4885	0.2470	0.7383	0.8092	0.2794	0.7712
17	26.81	21.20	25.08	19.33	27.49	27.49	19.69	29.78	0.6445	0.3088	0.5096	0.2922	0.8143	0.8227	0.3360	0.8388
18	23.30	20.83	23.43	19.84	23.17	23.14	19.55	26.95	0.6713	0.3787	0.5639	0.3933	0.6648	0.7747	0.3963	0.7646
19	24.27	20.00	23.82	18.06	23.91	23.85	18.99	27.83	0.6524	0.2954	0.5207	0.3127	0.6986	0.8016	0.3348	0.7913
20	24.96	21.15	24.84	18.69	24.98	24.76	16.59	30.66	0.6653	0.2753	0.5221	0.2478	0.7380	0.8318	0.2708	0.8546
21	24.54	20.16	23.70	17.76	23.66	24.15	19.48	27.94	0.6579	0.3253	0.5118	0.3315	0.5943	0.8170	0.3606	0.8237
22	23.15	19.86	23.13	18.71	22.10	22.65	19.33	28.38	0.6355	0.2539	0.4939	0.2960	0.5215	0.7678	0.3037	0.7641
23	19.28	19.41	21.12	17.47	22.07	18.96	18.84	30.94	0.5742	0.2065	0.3795	0.1864	0.5709	0.7587	0.2428	0.8714
24	24.79	20.36	23.91	19.61	24.42	25.24	19.06	26.50	0.6924	0.3585	0.5861	0.3963	0.7359	0.8228	0.3959	0.7950
Avg.	23.34	20.29	23.21	18.81	23.90	23.23	19.26	28.49	0.6490	0.3130	0.5114	0.3245	0.6572	0.7906	0.3466	0.8020
McMaster dataset																
	CPSNR								SSIM							
No.	CM1	CM2	CM3	CM4	CM5	CM6	CM7	PM	CM1	CM2	CM3	CM4	CM5	CM6	CM7	PM
1	16.54	19.46	19.19	19.74	20.59	16.15	17.09	24.08	0.5966	0.4867	0.5371	0.4583	0.5732	0.6265	0.4650	0.7191
2	20.73	21.58	22.07	19.95	22.87	20.10	19.18	28.06	0.6010	0.4209	0.5022	0.3632	0.5880	0.6816	0.3957	0.7877
3	22.29	20.49	22.32	19.87	22.85	22.20	18.98	25.75	0.7010	0.5153	0.6205	0.5129	0.7628	0.8070	0.5175	0.8282
4	24.53	20.44	23.32	20.00	25.80	25.49	17.50	27.94	0.7179	0.4464	0.6171	0.4752	0.9138	0.8833	0.4815	0.9034
5	21.06	20.64	22.50	19.78	22.94	20.52	18.78	27.34	0.6360	0.3744	0.5187	0.3807	0.6086	0.7314	0.3943	0.8081
6	21.21	20.82	22.64	19.59	23.68	20.65	19.35	27.60	0.6042	0.3592	0.4870	0.3428	0.6010	0.7054	0.3705	0.7908
7	23.19	21.06	23.77	19.99	26.72	23.37	19.34	28.26	0.6592	0.3750	0.5559	0.3862	0.7465	0.7656	0.3786	0.7660
8	26.86	23.86	25.52	19.78	29.03	26.96	20.19	29.73	0.6351	0.4812	0.5268	0.3050	0.8459	0.7972	0.3622	0.8207
9	19.49	20.91	21.74	18.76	22.29	18.82	18.43	28.67	0.5837	0.3675	0.4495	0.3257	0.5174	0.6915	0.3559	0.8365
10	17.58	20.84	20.37	19.71	21.08	17.15	18.28	28.89	0.5541	0.3884	0.4367	0.3331	0.4784	0.6315	0.3529	0.8135
11	18.00	21.15	20.69	19.44	22.54	17.53	18.47	29.91	0.4969	0.3507	0.3809	0.2806	0.4837	0.5586	0.3051	0.7948
12	18.65	20.62	21.41	17.73	23.67	17.92	18.16	29.78	0.5728	0.3018	0.3751	0.2600	0.4956	0.6600	0.2940	0.8558
13	20.55	20.02	22.09	16.67	24.22	19.22	18.44	32.29	0.5653	0.1968	0.3062	0.1631	0.4607	0.6944	0.2216	0.8820
14	19.67	20.98	21.79	18.83	23.60	19.09	19.01	30.98	0.5502	0.3041	0.3959	0.2348	0.5872	0.6683	0.2850	0.8344
15	17.86	21.14	20.40	18.87	21.94	17.19	18.10	30.98	0.5047	0.3322	0.3683	0.2247	0.4392	0.5859	0.2777	0.8161
16	15.87	18.06	18.50	21.53	20.10	15.49	17.23	26.50	0.5825	0.4662	0.5222	0.4982	0.5267	0.6143	0.4585	0.7429
17	16.60	19.80	18.78	20.16	20.83	16.08	17.44	24.38	0.5013	0.4300	0.4568	0.4032	0.5088	0.5389	0.4069	0.7004
18	19.16	20.64	20.91	20.31	21.49	18.76	18.08	28.17	0.6560	0.4417	0.5272	0.4333	0.5850	0.7335	0.4382	0.7988
Avg.	19.99	20.69	21.56	19.48	23.12	19.59	18.45	28.30	0.5955	0.3910	0.4769	0.3545	0.5957	0.6875	0.3756	0.8055

**Table 3:** Objective Performance Comparison in Average CPSNR (dB) and Average SSIM.

*Crosstalk Degradation 1 ($\sigma_{g}=0.45$ and $\sigma_{n}=0.01$)*								
	Kodak		McMaster		Set5		Set14	
Method	CPSNR	SSIM	CPSNR	SSIM	CPSNR	SSIM	CPSNR	SSIM
CM1	30.36	0.9363	26.45	0.8811	27.03	0.9060	27.09	0.8974
CM2	35.43	0.9218	34.57	0.9117	34.95	0.9164	33.17	0.8972
CM3	35.63	0.9276	34.54	0.9128	34.92	0.9186	33.21	0.9001
CM4	35.24	0.9314	34.53	0.9175	35.00	0.9233	33.06	0.9034
CM5	27.54	0.8676	28.66	0.8352	27.66	0.8575	27.51	0.8534
CM6	28.46	0.9229	24.90	0.8190	25.94	0.8801	24.61	0.8654
CM7	24.29	0.8950	22.07	0.8377	19.81	0.8353	19.90	0.7395
PM	36.69	0.9511	35.40	0.9386	35.42	0.9292	33.59	0.9098
*Crosstalk Degradation 2* ($\sigma_{g}=0.6$ and $\sigma_{n}=0.04$)								
	Kodak		McMaster		Set5		Set14	
Method	CPSNR	SSIM	CPSNR	SSIM	CPSNR	SSIM	CPSNR	SSIM
CM1	23.34	0.6490	19.99	0.5955	20.48	0.6541	20.66	0.6597
CM2	20.29	0.3130	20.69	0.3910	20.69	0.4302	20.14	0.4139
CM3	23.21	0.5114	21.56	0.4769	21.80	0.5260	21.50	0.5388
CM4	18.81	0.3245	19.48	0.3545	19.88	0.4105	19.50	0.4171
CM5	23.90	0.6572	23.12	0.5957	22.30	0.6064	22.11	0.5908
CM6	23.23	0.7906	19.59	0.6875	19.77	0.7383	20.12	0.7395
CM7	19.26	0.3466	18.45	0.3756	17.43	0.5693	18.04	0.4056
PM	28.49	0.8020	28.30	0.8055	28.67	0.8261	27.34	0.7879

## Data Availability

Not applicable.
